# Annotations

**Published:** 1897-08-21

**Authors:** 


### Annotations.

### Medical Dogmatism.

A well-told story, even if it be only one relating to a disease, is full of interest, and certainly the Retrospect of the Pathology of Pulmonary Phthisis which Dr. Burney Yeo lately gave to the Torquay Medical Society is not only of interest in itself as showing the extraordinary oscillations of opinion which may take place even within a generation, but teaches an important lesson in regard to medical dogmatism. It forms, in fact, a remarkable chapter in the history of medical pathology, a chapter which shows how dogmatic and assertive scientific men may become, and into how great errors they may fall when they trust too implicitly to certain favourite methods of investigation and follow too much their own preconceived notions. For instance, the inability to recognise the specific nature of tubercle, and the tendency to positively deny it, which so frequently appeared in the discussions of twenty-five years or so ago, had no real foundation in the thing itself. As Dr. Burney Yeo points out, pathologists rejected this very precise and definite view, which Laennec had asserted, and Villemin had gone far to establish, for such indefinite ideas as are to be gathered from the words "inflammation," "irritability," "irritation," "disposition," or "predisposition," and the like. But what in the shape of precise knowledge do such terms convey? What was there so fascinating in the word "inflammation" that it should have been adopted as able to cover all the facts in the life-history of phthisis? Why, again, was such great hostility manifested to the idea of specificity? It seems very difficult to find satisfactory answers to these questions, yet these errors and these tendencies were shared in by some of the most able and laborious pathologists of our time. With the new and exact methods of experimental research which we now possess, we seem able to arrive at conclusions more definite and more certain than were possible in those days; yet, even with those methods at our disposal, the history of the past—and not the very far past either—should teach us to exercise great caution against forming too hasty or too positive conclusions on subjects which present any scope for controversy. It is easy, even in scientific researches, to make false steps if every move be not subjected to rigid scientific proof.

### No Creed for the Criminal.

Some members of a Social Science Congress, which was held at Paris some time ago, visited a French prison, and after they had been shown through it one visitor asked to see the chapel. He was informed that there was none, and on pressing for an explanation of this, was told that the Government did not consider religious instruction to be necessary for criminals. In France, where agnosticism, like all else, goes uncompromisingly to its logical conclusion, one can understand this attitude; but one doubts if it is wise. For the mission of religion to the criminal is to teach him "just the meaning of his sorrow"; to explain to him why society takes upon itself to incarcerate him in a cell. He sees on one side himself and his desires; on the other society, his enemy, which frowns on his aims and punishes himself; religion should stand between to explain that law of regard for others, that limitation of the liberty of each for the benefit of all, on which our civilisation is based. To minds already intellectually trained this may be done by means of ordinary reasoning: but for the criminal, narrow-minded and often ignorant, the appeal to a Higher Power governing the world is necessary. The belief in a divine law will help to interpret the human laws. The method, as we have said, might not satisfy a close student of dogma; but as all the churches teach the fundamental truths of morality, it is better for criminals to learn the reason religion gives for being honest, patient, and self-controlled than to eat their heart out in rebellion against destiny and the law, ignoring their own share in bringing on their punishment.

### School Children as Wage-Earners.

A very interesting article with this title is contributed by Mrs. Hogg to the *Nineteenth Century*. The statistics do not show an absolutely large number of children who, although attending school, are employed at some trade in their leisure hours; but it shows a relatively large proportion who, from the age of eleven upwards, just when their education is becoming more intelligent, and they are more capable of storing it up for future use, are running errands, selling newspapers or doing some other task which tires them out, makes them languid and inattentive at lessons, without in the least putting them in the way of earning a living in the future. This is the saddest feature of it. Many employers complain of the Education Acts, because they prevent children from beginning to learn their future trade as early as they used to do; and, in fact, are killing out the system of long apprenticeships which, with all its hardships, used to make good workmen. No one listens to the protests of these gentlemen, and they are often enough based on sufficiently selfish grounds. But the employer who teaches him a trade is really a better friend to a child than the parent who lets him neglect school and schooling for the sake of a few pence a week, and a trade—to honour it by that name—which will teach him nothing, and may breed in him the idle, and often vicious, habits of the loafer. Mrs. Hogg shows that comparatively few of the children who have been thus occupied in their school days ever become skilled workmen, while a very large number drift permanently into the ranks of the unskilled. It may be pleaded that the parents who permit these side-tasks are poor, and are glad of an addition to the scanty family income. Where the child's wages amount to eighteenpence or half-a-crown, one might admit this plea. But these are the favoured few; and many parents allow their children to neglect their education and to work before and after school hours, and the whole of Saturday, for sixpence, twopence, or even a halfpenny a week. The last noble sum is frequently the remuneration bestowed on a girl for acting as nurse-housemaid to a neighbour. It may be said that in this case, at least, the child is learning something that will be useful in after-life. Let whosoever thinks this take a servant from the slums; then the cry will be that even a halfpenny was too much for such service as that poor little maid could give.

